# Oenological Potential of *Lachancea thermotolerans* and *Hanseniaspora uvarum* from High-Sugar Musts: Impacts on Fermentation and Wine Volatilome

**DOI:** 10.3390/microorganisms13102260

**Published:** 2025-09-26

**Authors:** María Trinidad Alcalá-Jiménez, Juan Carlos García-García, Juan Carlos Mauricio, Juan Moreno, Rafael A. Peinado, Teresa García-Martínez

**Affiliations:** Department of Agricultural Chemistry, Edaphology and Microbiology, Agrifood Campus of International Excellence ceiA3, Universidad de Córdoba, 14014 Córdoba, Spain; b52aljim@uco.es (M.T.A.-J.); p22gagaj@uco.es (J.C.G.-G.); qe1movij@uco.es (J.M.); qe1peamr@uco.es (R.A.P.); mi2gamam@uco.es (T.G.-M.)

**Keywords:** *Hanseniaspora uvarum*, *Lachancea thermotolerans*, killer phenotype, β-glucosidase activity, cellulase activity, aroma enhancement

## Abstract

Currently, there is little scientific data to support the importance of selecting non-*Saccharomyces* yeasts from different wineries in the Protected Designation of Origin (PDO) in Andalusia, southern Spain, and how this group of yeasts can affect the sensory properties of wine. Therefore, this research aimed to study some specific microbiological properties and the metabolites they could produce in order to evaluate the oenological potential of two non-*Saccharomyces* yeast strains isolated from a region of Andalusia (Córdoba, Spain), *Hanseniaspora uvarum* TJ-27 and *Lachancea thermotolerans* T-9, isolated from musts with high sugar content. Of 80 yeast isolates selected, these two strains were chosen for their notable β-glucosidase activity (observed in up to 40% of isolates), cellulase activity (present in 24%), and killer phenotype (found in 40%). In this study, strains that displayed characteristics associated with aroma release were selected. Fermentation assays using a high-sugar synthetic medium revealed that neither *H. uvarum* TJ-27 nor *L. thermotolerans* T-9 was able to complete alcoholic fermentation independently, achieving ethanol yields of only 5–6% *v*/*v*, indicating the need for subsequent fermentation by *Saccharomyces cerevisiae*. The originality of this study provides insight into the metabolites contributed by these strains to the wines produced. The best results were obtained when both strains were inoculated together. Furthermore, volatilome analysis showed elevated levels of key compounds such as isoamyl alcohols and 2,3-butanediol. These findings highlight the practical potential of using selected non-*Saccharomyces* strains from Andalusia to improve fermentation results and wine quality. The novelty of this study lies mainly in confirmation within region-specific isolates.

## 1. Introduction

The quality of wine is related to the microbial ecology of fermentation. *Hanseniaspora* has good oenological characteristics, providing a noticeable effect on the color, flavor, aroma, and stability of the wine [1]. *Hanseniaspora uvarum* (HU) (anamorph *Kloeckera apiculata*) is most prevalent in grapes, representing more than half of the yeast population during the grape harvest [2,3]. This species has a positive impact on the color, flavor, aroma, and stability of wine due to its significant role in the initiation of fermentation [2]. Furthermore, *H. uvarum* can increase the diversity of volatile compounds, thus improving the aromatic complexity of wine [4]. The aromatic complexity is due to the diversity of secondary metabolites and greater enzymatic activity [5]. *H. uvarum* produces and releases β-glucosidases in higher quantities than *Saccharomyces cerevisiae* [2].

Another important genus within the non-*Saccharomyces* is *Lachancea thermotolerans* or *Kluyveromyces thermotolerans*, as was previously known, was reassigned to the genus *Lachancea* based on the sequence analysis conducted by Kurtzman et al. [6].

*H. uvarum* has an ethanol tolerance of 3–5% (*v*/*v*) and is commonly used in mixed fermentations with *S. cerevisiae* to improve wine quality, as demonstrated in trials conducted by Hu et al. [7] and Borren and Tian [8]. Rementeria et al. [9] demonstrated that fermentations with a low predominance of *H. uvarum* species lack aromatic complexity.

*L. thermotolerans* (LT) is a ubiquitous yeast species present in numerous locations. Although it is most found on grapes, it can also appear in other habitats such as soil, insects, and plants [10,11]. This yeast species is found in spontaneous natural wine fermentations, with a low prevalence on days 2–4 of fermentation [12]. *L. thermotolerans* is capable of fermenting glucose and sucrose [13]. Regarding enzymatic activity, *L. thermotolerans* can express the following extracellular activities that influence wine aroma: esterase, esterase-lipase, β-glucosidase, pectinase, cellulase, xylanase, and glucanase [11]. *L. thermotolerans* has a moderate fermentative power, and several studies, such as those conducted by Gobbi et al. [14], Aponte and Blaiotta [15], and Morata et al. [11], confirm that it has tolerance to ethanol between 5 and 9% *v*/*v*. Furthermore, *L. thermotolerans* is a yeast that may produce 10% *v*/*v* of ethanol during fermentation [16]. This yeast ferments sugars into lactate and ethanol, redirecting the flow of carbon from ethanol to lactate. This leads to an increase in the production of lactic acid, a key attribute in wine aroma [17]. The various yeast species that develop during fermentation metabolize most components, primarily sugars. This gives rise to a wide range of volatile and non-volatile end products that influence the characteristics of the wine’s aroma and taste.

This study analyses the activity of β-glucosidase, an enzyme responsible for hydrolyzing substrates with glycosidic bonds and releasing glucose and aglycones. Under the catalytic action of β-glucosidase, these glucosides release volatile aromatic compounds, improving the sensory properties of wine. In addition to this enzyme, cellulolytic activity is also important, as it allows the degradation of cell wall polysaccharides, thus increasing the fruity and sweet aroma of wines due to the release of aromatic precursors. It also allows for improved clarification and filtration of the must [18].

This study aimed to evaluate the fermentation potential and metabolome of strains *H. uvarum* TJ-27 and *L. thermotolerans* T-9 in monoculture, coculture, and sequential culture, with a view to potential applications in oenology.

## 2. Materials and Methods

### 2.1. Isolation of Autochthonous Wine Yeasts

Yeast strains were collected from different wineries in the Montilla-Moriles Protected Designation of Origin (PDO) wine-growing region in southern Spain. This is a warm climate area in the province of Córdoba. These isolates were obtained from natural grape musts from two cooperatives: Cooperativa La Union (N37°34′47.657″, W4°38′13.818″) and Lagar Saavedra (N37°33′17.64″, W4°38′8.518″). A total of 80 isolates were recovered from both wineries (Supplementary Material Table S1). The aim was to isolate, characterize, and select native strains from this area. Those two strains showing the best microbial properties were then evaluated for their fermentation potential and contribution to the typicality of Andalusian wines.

### 2.2. Culture Media and Enzymatic Screening Procedures

#### 2.2.1. Wallerstein Laboratory Nutrient Agar (WLN Agar)

WLN Agar medium was prepared according to the specifications provided by Oxoid. The preparation involved dissolving 6% (*w*/*v*) of the dehydrated medium in distilled water, adding 2% (*w*/*v*) agar, and autoclaving at 120 °C for 15 min. After solidification in Petri dishes and incubation at 28 °C for three days, the medium enabled effective differentiation between yeast genera based on colony morphology and pigmentation, serving as a reliable visual identification method [18].

#### 2.2.2. Lysine Medium

To verify whether isolates were non-*Saccharomyces*, a selective lysine-based medium was used according to the protocol described by Alcalá-Jiménez et al. [18]. Isolates were confirmed as non-*Saccharomyces* (positive) if they exhibited growth after two consecutive subcultures on this medium.

#### 2.2.3. Determination of Yeast Killer Activity

The killer activity of yeast strains was assessed using methylene blue agar at pH 4.0, based on the method by Ramírez et al. [19] and Kaiser et al. [20]. Plates were first inoculated with 100 μL of a sensitive strain culture grown for 48 h. Test strains were then spotted on the surface and incubated in triplicate at 21 °C for seven days to observe potential killer activity.

#### 2.2.4. Medium for Detecting β-Glucosidase Activity

To evaluate β-glucosidase production, a qualitative assay was performed using a specific medium containing 0.5% (*w*/*v*) arbutin (Sigma-Aldrich Chemie GmbH, Taufkirchen, Germany), 0.1% (*w*/*v*) yeast extract (Oxoid, Basingstoke, UK), 1% (*v*/*v*) ferric chloride solution (Oxoid), and 2% (*w*/*v*) agar (Oxoid) [21]. After autoclaving the media at 120 °C for 15 min, they were inoculated and incubated at 28 °C for 14 days. This assay relied on the ability of β-glucosidase to hydrolyze arbutin, leading to observable color changes.

#### 2.2.5. Medium for Detecting Cellulase Activity

Cellulase activity was assessed by plating yeast on plates containing 1% (*w*/*v*) yeast extract (Oxoid), 2% (*w*/*v*) agar (Oxoid) (*w*/*v*), 2% (*w*/*v*) glucose (Oxoid), 2% (*w*/*v*) peptone (Oxoid), and 0.4% carboxymethylcellulose (CMC, Sigma). Plates remained at 30 °C for five days [22]. Colonies were washed with distilled water and stained with Lugol solution. Cellulase activity was considered as positive when a clear halo appeared around the colony.

#### 2.2.6. YPD Agar Medium

YPD (Yeast extract Peptone Dextrose) agar medium is composed of 1% (*w*/*v*) yeast extract (Oxoid), 2% (*w*/*v*) peptone (Oxoid), 2% (*w*/*v*) glucose (Oxoid), and 2% (*w*/*v*) agar (PanReac, Castellar del Vallès, Barcelona, Spain). It is the most widely used medium for developing and maintaining yeast growth, since it is rich in amino acids, nucleotide precursors, vitamins, and essential metabolites necessary for optimal cell growth.

In the alcoholic fermentation experiments, a modified version of YPD medium was employed but featured a notable adjustment: it incorporated a high sugar concentration of 20%, equally divided between glucose and fructose, to simulate the high sugar concentration of must.

### 2.3. Yeast Identification

Yeast identification was carried out using MALDI TOF (Matrix-Assisted Laser Desorption/Ionization Time-of-Flight Mass Spectrometry) Bruker Daltonics, ultraflextreme Bremen, Germany, at the Central Research Support Service of the University of Córdoba.

In the process of preparing the samples for analysis by MALDI-TOF-MS, the yeast was grown in YPD medium for short-term preservation and then dispensed into Eppendorf vials, in which 300 μL of Milli-Q water and 900 μL of absolute ethanol were added. The generated spectra were processed using the MALDI Biotyper Compass software version 4.1 (Bruker, Billerica, MA, USA), which calibrates the spectra and automates the measurement and identification procedures prior to result matching. The resulting spectra were then compared to reference profiles in the MBT Compass Library. Results are displayed using a color-coded system and a match score from 0 to 3, indicating the confidence of the identification. Red: No identification. Yellow: Identification at the genus level. Green: Identification at the genus and species levels. A score of 3 indicates a complete match, while a score of 0 means no match.

### 2.4. Alcoholic Fermentation Tests in Synthetic Media

As the final phase of the oenological characterization process for wine yeasts, an alcoholic fermentation test was performed to determine their fermentative power. The selection of these non-*Saccharomyces* yeasts is based on their potential to ferment, modulate, and improve the flavor, aroma, and clarity of wine, owing to their β-glucosidase and cellulase activities. However, these yeasts could not also be used as biological control agents, since they do not exhibit a killer phenotype.

In this study, fermentation tests were conducted with strains of different non-*Saccharomyces* yeast species, specifically *H. uvarum* TJ-27 and *L. thermotolerans* T-9.

The experimental conditions included monoculture, to assess the performance of each yeast strain individually under optimal conditions; coculture to evaluate their interactions when grown together; and sequential inoculation [23].

#### 2.4.1. Preparation of Pre-Inoculum

The yeast strains were seeded into 100 mL flasks containing liquid YPD medium and incubated with shaking at 100 rpm on a shaker (Infors ag ch-4103 bottmingen) for two days to promote optimal growth for the alcoholic fermentation trials. After incubation, the total number of cells in the pre-inoculum was determined using a Neubauer chamber (Paul Marienfeld GmbH&Co. KG, Lauda-Königshofen, Germany). This quantification was used to calculate the appropriate inoculation volume for each yeast strain, ensuring accurate inoculation across all cultures for the alcoholic fermentation experiments.

#### 2.4.2. Fermentation Monitoring 

Fermentations were carried out in 250 mL flasks containing 150 mL of modified YPD medium, with each flask inoculated to a final concentration of 2 × 10^6^ cells/mL. Three experimental conditions were tested, with three biological replicates of each. The first condition was monoculture of both strains individually, where the fermentative efficiency of each of these strains was assessed. The second condition was coculture (CO), where both strains were inoculated simultaneously at a 1:1 ratio. And the third condition was sequential culture (SQ). In the latter case, *H. uvarum* TJ-27 was inoculated first, since this genus of yeast is the first to appear in spontaneous fermentation, and on the fourth day, the *L. thermotolerans* T-9 strain was added. After inoculation, the flasks were incubated at 21 °C with slight and constant agitation. Fermentation kinetics was monitored by measuring the weight loss of each flask every two hours, following the method described by Spedding [24]. Fermentation was considered complete when no further weight loss was observed over several days. During each of the three fermentations carried out in the fermentation tests, the kinetics were monitored daily by weight loss of the flasks following the method described by Spedding [24]. Also, the yeast population in each culture was monitored throughout the fermentation process.

### 2.5. Determination of Microbiological Parameters

The total number of cells was determined from the appropriate dilution using a Neubauer chamber (Paul Marienfeld GmbH&Co. KG, Lauda-Königshofen, Germany). To monitor the yeast population in the different cultures daily, the number of viable cells was analyzed. For this analysis, 1 mL was first taken from each replicate, and serial dilutions were made. These cells were plated on WL agar to perform viable cell counts. Colony counts were performed after 48 h of incubation at 28 °C.

### 2.6. Determination of Oenological Parameters

Oenological parameters such as volatile acidity and residual sugar content were determined according to the official methods of the Organization International de la Vigne et du Vin (OIV) and the European Economic Community (EEC) [25]. Ethanol content was quantified using the dichromate oxidation method [26].

### 2.7. Analytical Determinations of Major Aroma Compounds

Major volatile compounds were quantified with a gas chromatograph HP 6890 Series II (Hewlett-Packard, Palo Alto, CA, USA) equipped with a capillary column with molten silica CP-WAX 57 CB (50 m in length, 0.25 mm in internal diameter, and 0.4 μm in coating thickness) and a flame ionization detector. Chromatographic conditions and sample preparation followed the procedures described by Peinado et al. [27]. Identification and quantification of the major volatile compounds were carried out by using standards submitted to the same treatment as the analyzed samples (see Supplementary Material).

### 2.8. Statistical Analysis

To identify differences among the different strains used, the parameters and aromatic compounds analyzed were subjected to statistical analysis using the Statgraphics Centurion statistical package (v. 16.1.11), MetaboAnalyst version 6.0 and Prism version 9.0 (GraphPad Software, La Jolla, CA, USA). All experiments were performed in triplicate. Quantified compounds were analyzed by simple ANOVA, and P tests were used to establish homogeneous groups (different letters indicate significant differences at a 95% confidence level). Moreover, principal component analysis (PCA) was conducted to further explore the data.

## 3. Results and Discussion

### 3.1. Microbiological Analysis

**Figure 1:**
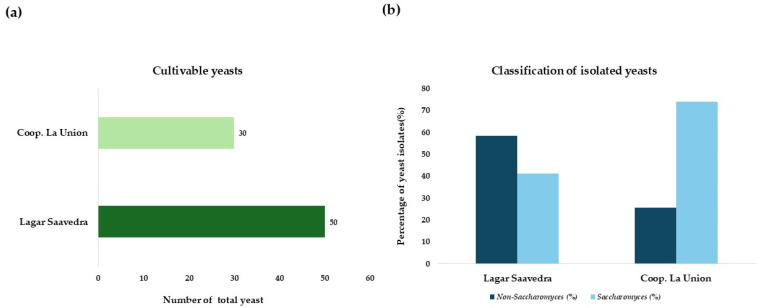
(**a**) Total number of isolates differentiated by wineries. (**b**) Number of isolates from each cooperative, differentiating between the percentage of *Saccharomyces* and non-*Saccharomyces*.

Based on the colony morphology in WL and lysine medium, a total of 80 yeasts were isolated from the two sources. The distribution of these isolates by winery is shown in Figure 1a. Figure 1b shows the number of isolates from each cooperative in percentage terms, broken down by *Saccharomyces* and non-*Saccharomyces yeasts*.

An analysis of the yeast populations across different winemaking environments revealed a higher proportion of non-*Saccharomyces* isolates in must from Lagar Saavedra, as depicted in Figure 1b. This finding is consistent with the fact that yeasts were isolated during spontaneous fermentation, as reported by Miranda et al. [28] and Alcalá-Jiménez et al. [23]. However, the highest percentage of isolates obtained at Coop. La Unión belonged to the *Saccharomyces* genus, when the theoretical alcohol content was above 8%. This is revealing, since it would indicate that the non-Saccharomyces yeast population in these wineries is displaced by the common genus, Saccharomyces. This observation aligns with the findings of Ruiz-Muñoz et al. [29], who reported that *S. cerevisiae* becomes the dominant species in the late stages of alcoholic fermentation.

**Figure 2:**
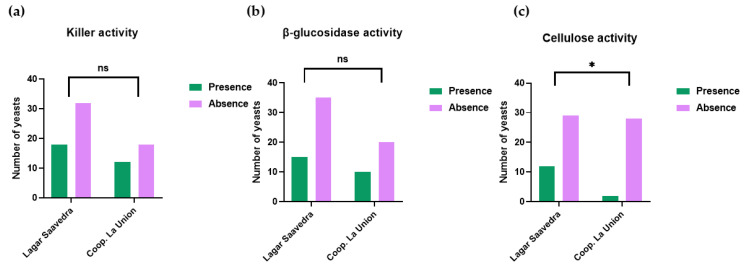
Functional phenotypes of yeast isolates in Montilla-Moriles Wineries (Córdoba, Spain). (**a**) Absence and presence of killer phenotype in yeast isolates from Montilla-Moriles (Córdoba, Spain). (**b**) Absence and presence of β-glucosidase activity in Montilla-Moriles yeast wineries (Córdoba, Spain). (**c**) Absence and presence of cellulose activity in Montilla-Moriles yeast wineries (Córdoba, Spain). Bar plots display the proportion of yeast isolates exhibiting each phenotype with significant differences: * means *q* < 0.05, ns means no significant differences.

One of the microbial properties attributed to yeasts is killing activity, which certain yeast strains exhibit. These killer substances have an antimicrobial function, inhibiting the growth and development of yeast strains that are susceptible to these toxins, but not their own producers. Figure 2 represents the microbial activities studied. The strains used as controls to evaluate the different activities were as follows: for killer activity, *Wickerhamomyces anomalus* was used as a positive control and a sensitive strain Ex33 *S. cerevisiae* as a negative control. For cellulose activity, *L. thermotelans* was used as a positive control and *S. cerevisae* as a negative control. For β-glucosidase activity, *Rhodotorula mucilaginosa* was used as a positive control and *T. delbrueckii* as a negative control.

Figure 2a represents the killer activity of various yeast isolates from spontaneous fermentation. This was assessed by determining the presence or absence of killer activity in each isolate. Killer activity is linked to natural biological control, as it can alter microbial population dynamics and thereby influence wine fermentation processes [23]. However, in this study, no significant differences in killer activity were observed between wineries. This suggests that the proportion of non-*Saccharomyces* and *Saccharomyces* yeasts is not influenced by this activity. Figure 2b shows the qualitative activity of the β-glucosidase enzyme observed in spontaneous fermentations of musts from different wineries in the Montilla-Moriles PDO. This enzymatic activity plays a crucial role in grape must, as some of the aromatic compounds exist in the form of non-volatile glucoside precursors that require β-glucosidase for their release and subsequent contribution to the aromatic profile of the wine [23]. Therefore, the presence of this activity favors the release of terpenes, phenylpropenes, and aliphatic esters [30]. This function contributes to the development of varietal aromas and wine typicity, also improving their organoleptic properties [31,32]. Consequently, as seen in Figure 2b, it can be concluded that the yeasts from both wineries have a high potential to enhance the aromas associated with glucoside-derived compounds. Figure 2c shows the cellulolytic activity of the different isolates in musts of spontaneous fermentations carried out at wineries in the Montilla-Moriles PDO. Cellulolytic activity is responsible for degrading cellulose, thereby improving the clarification and filtration of produced wine [33,34]. A significant difference is observed between the isolates from both wineries. This could indicate that the spontaneously fermented yeasts isolated from Lagar Saavedra exhibit greater cellulite activity than the yeasts isolated from Coop. La Unión.

### 3.2. Yeast Identification and Selection

The non-*Saccharomyces* yeast species identified in this study included *L. thermotolerans* (five strains) and *Hanseniaspora opuntiae* (two strains) from Coop. La Union and *Torulaspora delbrueckii* (three strains), *H. uvarum* (one strain), and *Pichia kudriavzevii* (two strains) from Lagar Saavedra. These strains were chosen from among all identified species because they exhibited at least one of the microbial properties described in Section 2 (Materials and Methods). Specifically, only strains that showed cellulose and β-glucosidase activities were selected for further analysis in this study. These strain species were *H. uvarum* TJ-27 from Coop. La Union and *L. thermotolerans*-T9 from Lagar Saavedra. The related study by Gonzalez-Alonso [35] supports our results, where it was shown that *L. thermotolerans* species show cellulase activity and, to a lesser extent, *H. uvarum*. Regarding β -glucosidase activity, in this study, the strains did not show this activity; however, these results agree with the results obtained by Muradova et al. [36], where *H. uvarum* showed a high production of this enzyme or in the study carried out by Morata et al. [11], where it is shown that *L. thermotolerans* presents said activity.

### 3.3. Fermentation Kinetics

**Figure 3:**
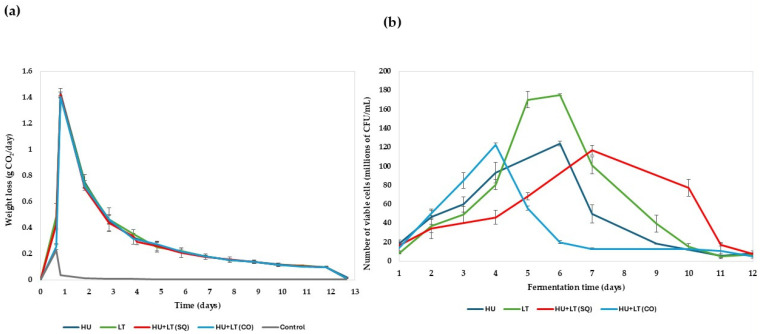
(**a**) Kinetic profiles of different fermentations performed with *H. uvarum* TJ-27(HU) and *L. thermotolerans* T-9 (LT) in monoculture. In sequential (SQ), where *H. uvarum TJ-27* was added first, and on the fourth day, *L. thermotolerans* T-9 was introduced and cocultured (CO). (**b**) Viable cell count under the different fermentation conditions performed with *H. uvarum* TJ-27 (HU) and *L. thermotolerans* T-9 (LT), as described in the previous figure. The means and standard deviations are represented.

Table 1 presents the general oenological parameters. Figure 3a shows the kinetic profiles obtained during daily monitoring of each of the fermentations carried out with the yeast *H. uvarum* TJ-27 in monoculture (HU) and *L. thermotolerans* T-9 in monoculture (LT) and in mixed culture, as performed in the study by Vaquero et al. [16], where both yeast genera were used for kinetic study. This graph also shows the kinetics of the co-inoculation (CO) and sequential (SQ) fermentations. These exhibited similar behavior to monoculture fermentation. It is worth noting that the maximum CO_2_ release was obtained approximately 24 h after the start of fermentation in both cases. From that moment on, the fermentation rate gradually decreased to practically zero, marking the end of this process for the yeasts used in the trial. In addition to the daily monitoring of the kinetic profiles of the fermentations, the population growth of the different yeast strains was monitored daily during this process (Figure 3b). This study of population growth allows us to determine whether there is a relationship between fermentation kinetics and the yeast population present in the medium.

In the case of monoculture fermentations, inoculated with only one of the two yeast strains analyzed, according to the daily total cell count, the *L. thermotolerans* T-9 strain showed a significantly higher growth rate as the fermentation progressed than the *H. uvarum* TJ-27 strain. Furthermore, the viability of *H. uvarum* TJ-27 was lower than that of *L. thermotolerans*. On the other hand, in mixed fermentations, specifically under CO, the evolution of the yeast population, expressed in terms of the number of viable cells, was greater during the first hours, coinciding with the maximum amount of CO_2_ released, suggesting that their behavior is not affected. However, as seen in Table 2, this coculture exhibits higher values of interesting aromatic compounds than monoculture fermentations, such as isobutanol, 2-phenylethanol, and isoamyl alcohols. However, in the sequential fermentation, maximum viability lasted longer, rather than showing a prominent peak on a specific day, compared to the other fermentations performed with *H. uvarum* TJ-27 or *L. thermotolerans* T-9 strains. These results are very interesting, since inoculating the *H. uvarum* TJ-27 strain first and then the *L. thermolerans* strain could be considered a strategy to reduce the ethanol content of alcoholic fermentation, as can be seen in Table 1.

### 3.4. Oenological Parameters

The two non-*Saccharomyces* yeast species analyzed showed similar ethanol contents (≈5–6% *v*/*v*) in both the simple *H. uvarum* TJ-27 fermentation and sequential cultures. However, *L. thermotolerans* T-9 monoculture had an ethanol content of 7% *v*/*v*, a significant difference compared to the other cultures. It was observed that sequential culture showed a significant difference compared to coculture. This could be due to the addition of *L. thermotolerans* T-9 to terminate the fermentation, which caused their ethanol level to decrease. As observed in the residual sugar values, these yeasts were unable to complete the fermentation, so it would be advisable to terminate this fermentation with *S. cerevisiae*.

Wine quality depends on multiple factors, one of the most important being volatile acidity, whose legal limit is 1.2 g/L for most wines. However, it is advisable to keep it as low as possible, since acetic acid can be sensorially detected by the consumer at concentrations above 0.8 g/L and confer an undesirable vinegary taste and aroma to the final product. As observed, in monoculture and mixed cultures, the presence of *L. thermotolerans* T-9 can cause an increase in volatile acidity, as also confirmed in the study carried out by Sánchez-Suárez et al. [37]. Regarding the rest of the chemical parameters, there are no significant differences between the different conditions.

### 3.5. Major Aroma and Statistical Analysis

**Figure 4:**
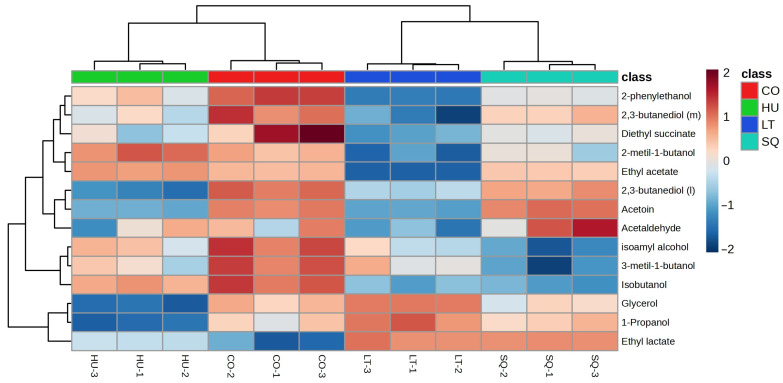
Representation of the heat map of the quantification data of the normalized major aroma compounds of the four different wines: wine with both strains in coculture, CO (red), wine with *H. uvarum* TJ-27 (HU) strain (green), wine with *L. thermotolerans* T-9 (LT) strain (blue), and wine with the strains inoculated sequentially, SQ (turquoise). Means: l = Levo, m = Meso. The highest concentration is assigned red, and the lowest concentration is blue.

Figure 4 presents a heat map of the quantification data of the normalized major aroma compounds of the four different wines (Supplementary Material Table S2). This image shows that the triplicates of each wine are more similar to each other than to the rest of the samples. In wine with the *L. thermotolerans* T-9 strain, a high quantification of the compound’s glycerol, ethyl lactate, and 1-propanol is observed. In wine with the *H. uvarum* TJ-27 strain, large quantifications were detected for two compounds, ethyl lactate and ethyl acetate. In wine with cocultivated strains, high quantifications were found in the compounds 2,3-butanediol (*levo*), 2-phenylethanol, 2,3-butanediol (*meso*), isoamyl alcohol, diethyl succinate, and isobutanol. In the wine inoculated by both strains sequentially, the quantifications of the compounds acetoin and acetaldehyde stood out.

All the alcohols determined in this study, except for propanol, whose precursor is α-ketobutyric acid, are derived from the degradation of sugars or amino acids through the decarboxylation of keto acids. These products contribute solvent-like aromas to the wine, except for 2-phenylethanol, which has a floral aroma. Its overall concentration should not exceed 400–500 mg/L. Below these levels, they contribute to the wine’s aromatic complexity [38].

In Table 2, isoamyl alcohols show significant differences under the conditions tested. The highest concentrations were observed with coculture and monocultures of both strains, while the lowest concentrations were achieved in the sequential culture. Isobutanol and 2-phenylethanol exhibit similar behaviors. The lowest values are found in *L. thermotolerans* T-9 monoculture and in the sequential assay, where *L. thermotolerans* T-9 was added on the fourth day, leading us to conclude that *L. thermotolerans* T-9 is capable of lowering the production of these compounds. 2-phenylethanol provides sweet and floral aromas, as observed in the study by Gómez-Míguez et al. [39]. In our study, only two of the four conditions passed the perception threshold (10 mg/L).

The highest values were observed in the co-inoculation and *H. uvarum* TJ-27 monoculture, while the lowest values were found in the sequential assay and in the *L. thermotolerans* T-9 monoculture. In the study conducted by Martin et al. [5], it was observed that when 2-phenylethanol increased, the floral and sweet attributes of the wine were intensified, which concluded that our strains in coculture and in *H. uvarum* TJ-27 monoculture could be used to intensify the varietal aromas of typical Andalusian wines.

Among the carbonyl compounds, acetaldehyde is formed by yeasts as an intermediate metabolite in the conversion of glucose to ethanol. Acetaldehyde has a pleasant, fruity aroma at minimal concentrations. However, at high concentrations, its odor becomes unpleasant [23]. Under all conditions, the amount is higher, so these yeasts must have high pyruvate decarboxylase activity.

In this study, it was observed that the compounds 2,3-butanediol (*levo*) and glycerol were produced at lower levels in the *H. uvarum* TJ-27 monoculture. This could be because the selected strain has lower glyceropyruvic fermentation activity than *L. thermotolerans* [40].

The elevated levels of acetaldehyde and acetoin are likely due to redox balance, where a shift in their metabolism occurs toward glyceropyruvic fermentation to regenerate the NAD^+^ that could not be obtained through alcoholic fermentation.

Acetoin contributes to the development of lactic acid odors. Under the test conditions, the perception threshold was exceeded, and higher values were detected in mixed cultures. However, in axenic cultures, a significant reduction in this compound was observed compared to mixed cultures.

On the other hand, the elevated levels of acetaldehyde (129–194 mg/L in the different cultures) are likely the result of elevated pyruvate decarboxylase activity in these non-*Saccharomyces* strains, exceeding typical wine concentrations (50–70 mg/L). At these concentrations, both acetaldehyde and acetoin can contribute to spicy and oxidized apple aromas as well as lactic acid odors, adding character to the wine.

In these fermentations, lactic acid is produced, which translates into ethyl lactate. This is because the increase in ethyl lactate is associated with high levels of lactic acid during fermentation, as described by Guo et al. [41]. As seen in Table 2, the highest concentrations of this compound are found in the monoculture of *L. thermotolerans* T-9 and in the co-inoculation. *L. thermotolerans* T-9 provides fruity aromas; since it produces fruity esters such as ethyl lactate, this agrees with the results obtained by Bartolomé et al. [42] and Muñoz-Castells et al. [17]. However, we see that in the monoculture of *H. uvarum* TJ-27 and in the sequential culture, where it was first inoculated with *H. uvarum*, a lower concentration of this ester is observed, so it can be concluded that, in the presence of *H. uvarum*, a reduction in lactic acid occurs.

The highest concentrations of diethylsuccinate were detected in *L. thermotolerans* T-9 monocultures and cocultures. As reported in the study by Delač Salopek et al. [43], *L. thermotolerans* T-9 can increase the production of this compound, contributing fruity notes to the wine compared to pure *H. uvarum* TJ-27 or SQ cultures, where this compound is present at a lower concentration.

It was also shown that, in the presence of *L. thermotolerans*, ethyl acetate concentration tends to decrease, as observed in the sequential culture or monoculture of this strain, which presents the lowest values in all conditions. Ethyl esters are very interesting because they have a pleasant fruity and floral aromatic note [44]. A higher production of this compound was observed in the monoculture of *H. uvarum*, or in the coculture; this is because the bee-feeding yeasts are good producers of acetate esters, as observed in the study of Rojas et al. [44] and Alcalá-Jiménez et al. [23].

Our study is supported by the results obtained by Benito-Castellano et al. [45] and del Fresno et al. [46], where these species can increase aromatic complexity. These authors also describe that *L. thermotolerans* T-9 decreases ethanol content, which is also observed in our study, concluding that this strain is capable of helping mitigate the effects of climate change.

In the study carried out by Liao et al. [47], it is explained that a higher expression of the β-glucosidase activity of *H. uvarum* increased the contents of 2-phenylethanol or isoamyl alcohols. Therefore, a higher expression of this enzyme is related to an increase in these compounds, supporting the data obtained in our research between the two strains in pure cultures, as indicated in Table 2. However, it is observed that in co-inoculated cultures, these compounds have better values. This is supported by the published studies of Gallo et al. [48], Zhang et al. [49], or Yang et al. [50], where the best results were obtained in mixed cultures.

In the study carried out by Alcalá-Jiménez et al. [18], it was observed that those wines whose microbiota showed greater cellulose activity had a higher concentration of compounds associated with fruity aromas, as found in this research.

**Figure 5:**
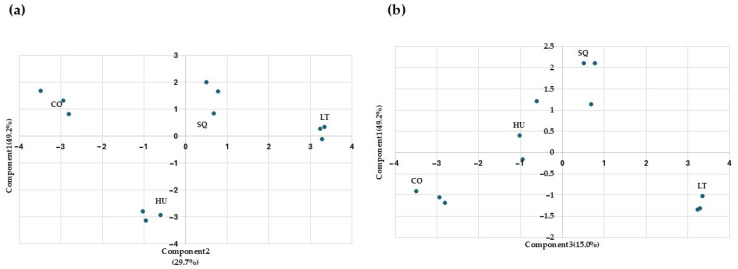
Principal component analysis of the “wines” obtained after fermentation kinetics under different conditions: *H. uvarum* TJ-27 (HU) and *L. thermotolerans T-9* (LT) in monoculture. In sequential (SQ), where *H. uvarum* TJ-27 was added first and on the fourth day, *L. thermotolerans T-9* was introduced and cocultured (CO). The analysis was performed on the major volatile compounds. (**a**) Principal component analysis between components one and two. (**b**) Principal component analysis between components one and three.

With the aim of reducing the number of variables, a principal component analysis was carried out (Figure 5). Components are a lineal combination of the input variables, and the interpretation of the components obtained must be conducted by the analyst. Here, the aroma compounds have been selected as input variables. The first three components explain 93.9% of the observed variability (Figure 5a,b). Component 1 differentiates *L. thermotolerans* T-9 and *H. uvarum* TJ-27 assay from the rest. The compounds with the greatest influence are propanol, 2-phenylethanol, and ethyl lactate. Component 2 differentiates co-inoculation from SQ and *L. thermotolerans*. This component is mainly influenced by 2,3-butanediol (*levo*), 1-Propanol, and acetoin. Lastly, component 3 differentiates SQ from CO, and the most influential compounds are acetaldehyde and isoamyl alcohol.

## 4. Conclusions

This study highlights the significant potential of non-*Saccharomyces* yeasts, specifically *Hanseniaspora uvarum* and *Lachancea thermotolerans*, isolated from complex enological environments, to enhance wine quality through their marked β-glucosidase and cellulase activities. These enzymatic properties are essential for releasing aromatic and clarification-promoting compounds, making these strains promising candidates for controlled fermentations.

Our findings indicate that *H. uvarum* TJ-27 and *L. thermotolerans* T-9 are particularly proficient at augmenting aroma complexity and improving the overall sensory profile of wines by facilitating the release of varietal aromas. The best results were obtained with co-inoculation. Nevertheless, since these strains are unable to complete fermentation independently, their use should be complemented by *Saccharomyces cerevisiae* to ensure a successful fermentation process.

## Figures and Tables

**Table 1:** Oenological parameters. Values shown represent averages of triplicate samples (data are mean ± standard deviation (SD)). Concentrations with different superscript letters in the same row are significantly different (*p* < 0.05).

Oenological Parameters	*H. uvarum*	*L. thermotolerans*	Co-Inoculation	Sequential
Volatile acidity (g/L)	0.176 ± 0.01 ^a^	0.349 ± 0.04 ^a^	0.332 ± 0.01 ^a^	0.343 ± 0.08 ^b^
Reducing sugar(g/L)	95.7 ± 1.6 ^c^	70.83 ± 3.4 ^a^	83.2 ± 2.5 ^b^	100.5 ± 0.00 ^d^
Ethanol (%, *v*/*v*)	5.20 ± 0.02 ^a^	7.00 ± 0.02 ^c^	6.20 ± 0.01 ^b^	5.30 ± 0.02 ^a^

**Table 2:** Major aroma compounds. The values shown represent averages of triplicate samples (data are mean ± SD). Values with different superscript letters in the same row are significantly different test (*p* < 0.05).

Compounds (mg/L)	*H. uvarum*	*L. thermotolerans*	Co-Inoculation	Sequential (SQ)
Alcohols				
1-Propanol	7.0 ± 0.3 ^a^	16.4 ± 1.2 ^c^	12.6 ±1.1 ^b^	13.3 ± 0.8 ^b^
Isobutanol	21.3 ± 0.6 ^b^	15.0 ± 0.7 ^a^	24.0 ± 1.2 ^c^	14.4 ± 0.6 ^a^
Isoamyl alcohols	46.3 ± 3.1 ^b^	42.8 ± 2.7 ^b^	54.5 ± 2.5 ^c^	35.2 ± 2.4 ^a^
2-phenylethanol	11.5 ± 1.9 ^b^	4.8 ± 0.1 ^a^	21.0 ± 1.7 ^c^	9.9 ± 0.2 ^b^
Carbonyl Compounds				
Acetaldehyde	174.8 ± 15.3 ^a^	160.1 ± 5.6 ^ab^	183.1 ±12.1 ^ab^	193.7 ± 15.6 ^b^
Acetoin	145.8 ± 5.9 ^a^	132.7 ± 5.5 ^a^	155.0 ± 2.0 ^b^	156.0 ± 1.0 ^b^
Esters				
Ethyl lactate	9.8 ± 0.5 ^a^	70.4 ± 7.0 ^b^	64.7 ± 2.0 ^b^	14.0 ± 0.1 ^a^
Diethyl succinate	126.0± 1.0 ^a^	134.2± 2.0 ^b^	133.2± 3.0 ^b^	128.0± 3.0 ^a^
Ethyl acetate	8.9 ± 0.8 ^d^	0.0± 0.0 ^a^	5.3 ± 0.3 ^c^	3.8 ± 0.3 ^b^
Polyols				
2,3-Butanediol (*levo*)	47.4± 4 ^a^	69.3 ± 2.7 ^b^	137.9 ± 7 ^d^	117.8 ± 6 ^c^
2,3-Butanediol (*meso*)	47.1 ± 3.9 ^b^	34.4 ± 4.4 ^a^	64.0 ± 5.0 ^c^	53.5 ± 2.0 ^b^
Glycerol (g/L)	1.90 ± 0.06 ^a^	3.9 ± 0.01 ^d^	3.20 ± 0.1 ^c^	2.90 ± 0.16 ^b^

## Data Availability

The original contributions presented in this study are included in the article/Supplementary Material. Further inquiries can be directed to the corresponding author.

## Supplementary Materials

The following supporting information can be downloaded at https://www.mdpi.com/article/10.3390/microorganisms13102260/s1, Supplementary Material Table S1: Yeast characterisation; Supplementary Material Table S2: Volatile compounds.
